# An in vitro assessment of the residual dentin after using three minimally invasive caries removal techniques

**DOI:** 10.1038/s41598-024-57745-0

**Published:** 2024-03-26

**Authors:** Rand Mohammed Al-Sagheer, Ali J. Addie, Lamis A. Al-Taee

**Affiliations:** 1 Department of Conservative and Aesthetic Dentistry, Baghdad College of Dentistry, University of Baghdad, Baghdad, Iraq; 2 Centre of Advanced Materials, Ministry of Science and Technology, Baghdad, Iraq

**Keywords:** Biotechnology, Medical research

## Abstract

To evaluate the efficiency and effectiveness of three minimally invasive (MI) techniques in removing deep dentin carious lesions. Forty extracted carious molars were treated by conventional rotary excavation (control), chemomechanical caries removal agent (Brix 3000), ultrasonic abrasion (WOODPECKER, GUILIN, China); and Er, Cr: YSGG laser ablation (BIOLASE San Clemente, CA, USA). The assessments include; the excavation time, DIAGNOdent pen, Raman spectroscopy, Vickers microhardness, and scanning electron microscope combined with energy dispersive X-ray spectroscopy (SEM–EDX). The rotary method recorded the shortest excavation time (p < 0.001), Brix 3000 gel was the slowest. DIAGNOdent pen values ranged between 14 and 18 in the remaining dentin and laser-ablated surfaces recorded the lowest reading (p < 0.001). The Ca:P ratios of the remaining dentin were close to sound dentin after all excavation methods; however, it was higher in the ultrasonic technique (p < 0.05). The bur-excavated dentin showed higher phosphate and lower matrix contents with higher tissue hardness that was comparable to sound dentin indicating the non-selectiveness of this technique in removing the potentially repairable dentin tissue. In contrast, the MI techniques exhibited lower phosphate and higher organic contents associated with lower microhardness in the deeper dentin layers. This was associated with smooth residual dentin without smearing and patent dentinal tubules. This study supports the efficiency of using MI methods in caries removal as conservative alternatives to rotary excavation, providing a promising strategy for the clinical dental practice.

## Introduction

The management of deep dentin carious lesions have been directed towards more conservative and biological directions^1^, as the traditional rotary excavation causes adverse biological reactions to the dentin-pulp complex. This is attributed to the heat and vibration associated with the procedure, as well as the non-selective behavior in removing both healthy and diseased tissues^2,3^.

Finding effective minimally invasive (MI) techniques to overcome the drawbacks of bur excavation is an objective for dental researchers, in order to remove the superficial, bacterially-contaminated and denatured dentin while preserving the partially demineralized tissue that can be sealed with suitable therapeutic restorations^4^. This will maintain the integrity of healthy and mineralizable tooth tissue, while maximizing the reparative potential of dentin-pulp complex^5^. These methods include laser ablation^6^, air abrasion^7^, sono-abrasion^8^, and the chemomechanical agents (CMCR)^9^.

BRIX 3000 (Brix medical science, S.R.L. of Argentina) is a papain-based gel with high enzyme activity (3.000 U/mg) due to utilizing the encapsulation buffer emulsion technology, that confers the stability of enzyme and thus reactivity^10^. The chemical method is considered to be effective in dissolving the infected tissues facilitating their removal while preserving the sound dentin structure^11^.

The ultrasonic abrasion can remove carious lesions and unsupported hard structure when used at high frequency (20–40 kHz). It relies on the kinetic energy of water molecules that transferred into tooth surface by high-speed oscillated diamond-coated cutting tip accompanied with sonic air-scaler device^12^. However, Neves et al. (2011)^11^ found that the sonic technique by using tungsten-carbide tips (Cariex system) is efficient to remove the caries at the cavity floor in a micro-CT study while still residual caries left at the walls, which agreed with a previous study that revealed a tendency towards under preparation with sono-abrasion^13^. However, the utilization of advanced systems with different parameters might showed different caries removal efficiency.

Erbium-doped Yttrium Aluminum Garnet (Er: YAG), and Erbium, Chromium-doped Yttrium, Scandium, Gallium and Garnet (Er, Cr: YSGG) lasers have demonstrated promising results in caries removal when proper parameters are utilized. Both lasers transfer high energy into dental hard tissues through a photoablative process mediated by a rapid expansion of subsurface water, leading to explosive ejection of tissue from the surface^14^. A previous study^11^ that applied Er:YAG laser equipped with a laser-induced fluorescence (LIF) feedback system reported a limited minimally-invasive potential of this laser, due to excessive removal of inactive and stained carious tissues resulted in over-excavation. Whilst, Kinoshita et al. (2003)^15^ supported the efficiency of using Er,Cr:YSGG laser in caries removal, as it produced smooth and wavy residual dentin surfaces with little smearing in comparison to the rotary and chemomechanical caries excavations.

Raman spectroscopy, is a non-invasive quantitative technique that can chemically characterize healthy and carious dentin through their specific molecular vibrational energy signatures^16,17^. It can provide a molecular information regarding the mineral and matrix components of biologically compromised tissues^18^. The integrity of collagen triple helix in type I collagen membrane was previously measured^17,19^ in which the peak ratio of amide III to C-H bond of the pyrrolidine ring (1235 cm^−1^ :1450 cm^−1^) indicates the integration of the collagen if it is close to 1. The microhardness remains the gold standard measure for the mineral contents of dental hard tissues and the enhanced hardness reflects better crystalline and denser apatite structures^20^.

Therefore, this study compared the efficiency of three minimally invasive caries removal techniques (Brix 3000, ultrasonic abrasion, and Er,Cr:YSGG laser ablation) in comparison to the conventional rotary excavation (control). The assessments included excavation time, along with biochemical, mechanical, and morphological properties of the residual dentin examined through Raman spectroscopy, Vickers microhardness testing, and scanning electron microscopy with energy dispersive X-ray spectroscopy (SEM–EDX), respectively. The proposed null hypothesis was; these MI methods wouldn’t show a selectivity in caries removal in comparison the non-selective conventional rotary method.

## Methods

### Sample preparation

**Figure 1:**
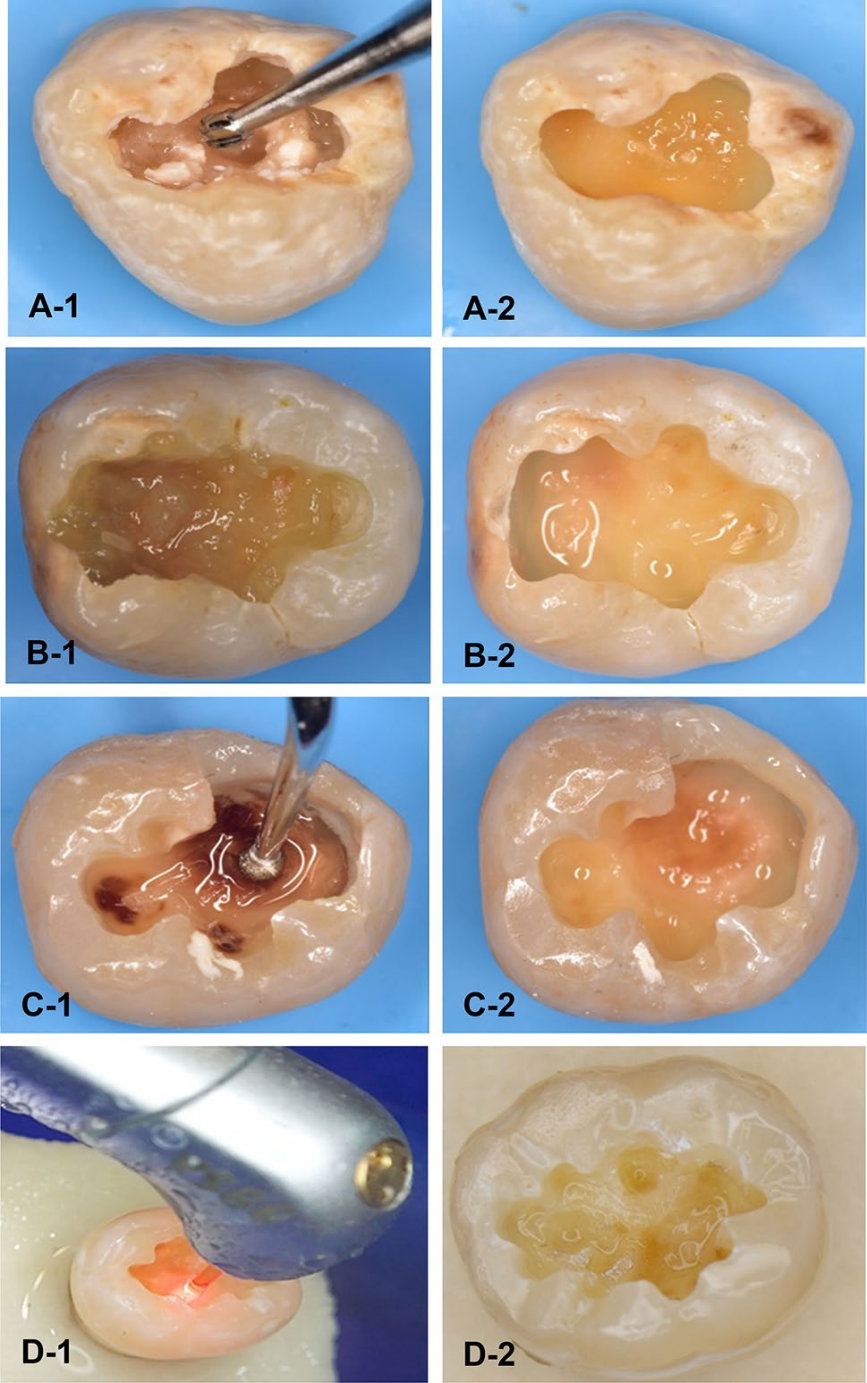
The caries excavation techniques for removing deep carious dentin lesions. (**A-1**) rotary excavation, (**B-1**) Brix 3000 application, (**C-1**) Ultrasonic Caries Removal Tip SB1, (**D-1**) Waterlase iPlus Er,Cr:YSGG Laser. (**A-2**,**B-2**,**C-2**,**D-2**) The residual dentin after each technique, respectively.

**Figure 2:**
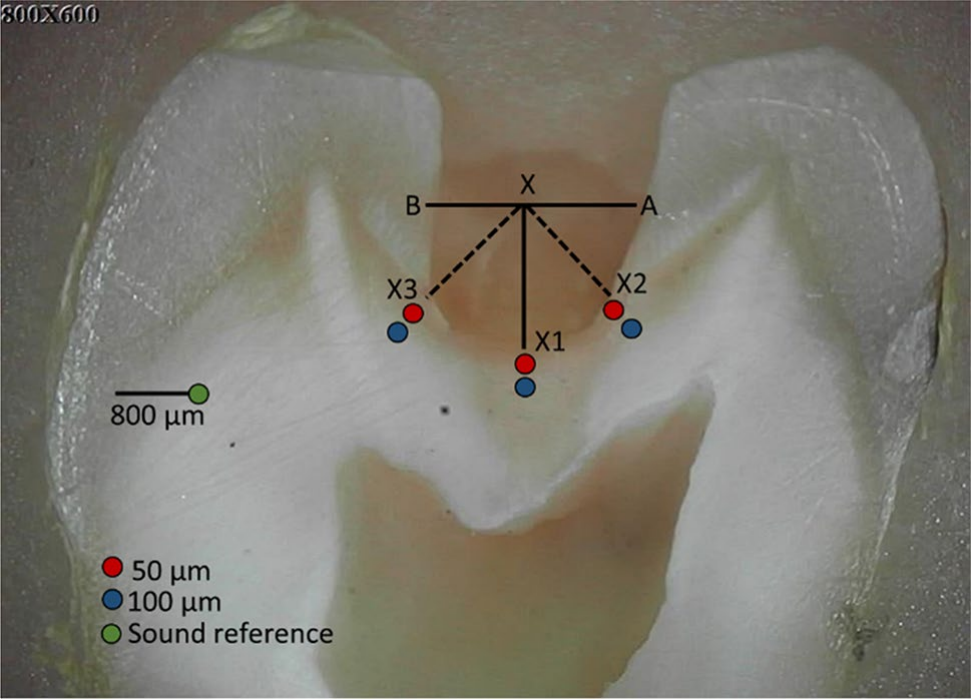
The selected points at the excavated margin and the sound reference point (800 µm from the DEJ) that were examined by Raman spectroscopy followed by Vickers microhardness.

Forty human carious molars were collected from patients between 25 and 40 yrs. for periodontal reason using an ethics protocol approved by the health research committee (Ref No. 754, 28/12/2022). In which the natural caries extends through dentin without pulp exposure (score 4 following the International Caries Detection and Assessment System (ICDAS-II)^21^. An access cavity preparation was prepared through enamel in each tooth (2 mm depth) using tungsten carbide bur (Komet, Lemgo, Germany) at a high-speed air turbine handpiece (300 k rpm) to expose the carious lesion in dentin. In the 1st group (Rotary excavation, n = 10), the carious lesion was removed by a tungsten- carbide round bur (size #12, Komet, Lemgo, Germany) in a slow-speed handpiece with circular light brush strokes (Fig. 1A-1,A-2). In the 2nd group, the caries was removed chemomechanically by the application of Brix 3000 (Brix S.R.L., Area Industrial, Los Arces, Santa Fe, Argentina) following the manufacture’s instruction. The gel was dispensed into the cavity and agitated immediately using non-sharp spoon excavator hand instrument (TGA Reg DENSOL, Ar Instrumed, Australia, 1.3 mm diameter). After 2 min the cavity scraped away using the blunt end of excavator in a pendulum movement without pressure until a clinically hard cavity surface was obtained. The gel was washed away periodically prior to check the hardness of the remaining dentin with a sharp probe (EXD17-23, Osung, USA). This procedure was repeated two to three times until the soft caries was completely removed (Fig. 1B-1,B-2)^22^.

In 3rd group (Ultrasonic abrasion), the soft caries was removed using the diamond-coated tips SB1 (WOODPECKER, EMS Type, GUILIN, China, 1.5 mm diameter) attached to WOODPECKER air-scaler handpiece (UDS-A LED), and ultrasonic dental scaler (WOODPECKER UDS-A LED, GUILIN, China). The vibration excursion was ≤ 100 μm with 28 ± 3 kHz vibration frequency following manufacturer’s instructions. The procedure was done using copious water until hard dentin was reached (Fig. 1C-1,C-2). The carious tissues, in the 4th group were removed by a WATERLASE iPlus Er,Cr:YSGG Laser (WATERLASE iPlus, BIOLASE Technology, Inc., California, USA) with Quartz tips (MZ- 8, 800 µm diameter, and 6 mm length) attached to a contra-angle gold handpiece (BIOLASE, Inc., California, foothill Ranch, USA). The wavelength of the laser is 2780 nm used in H mode, 15 Hz frequency, 3 W power output, < 150 mJ energy value. This was accompanied with copious amount of water as recommended by the manufacturer. Caries was excavated by moving the fiber tip gently in a circular or brushing motion in and-out motion, in a1.5 mm distance away from the cavity (a non-contact method)^23^ (Fig. 1D-1,D-2).

The excavation endpoint was determined when the remaining dentin appeared yellow to light brown upon visual inspection. Tactile probing provided further assessment, with firm or leathery/scratchy sensation indicating probable sound or caries-affected dentin, respectively^24,25^. The surfaces were then examined using a DIAGNOdent pen (655 nm diode laser, KaVo, Germany) calibrated per manufacturer’s guidelines. Sound site fluorescence provided a baseline reading for each cavity. The mean of three readings across each cavity was calculated, with values between 0 and 20 denoting probable sound or caries-affected dentin^26^. Excavation times were recorded in minutes by digital stopwatch, incorporating time for washing, drying, and re-inspection of cavity surfaces. For standardization, all procedures were conducted by the same operator.

After caries excavation, each tooth was hemi-sectioned longitudinally in a mesiodistal direction (Isomet 1000, Buehler, Lake Bluff, IL, USA) through the prepared surface with water-cooled diamond blade (330-CA/RS-70300, Struers, Detroit Rd. OH, USA). The buccal halves were mounted in epoxy resin molds exposing the prepared surfaces that kept hydrated throughout the experiments. To get flat and smooth surfaces, the assessed substrates received a sequential polishing (P1200 for 10 s, P2500 for 10 s and P4000) followed by 10 min ultrasonic cleaning^17^.

### Raman spectroscopy

A high-resolution confocal Raman microscope (Senterra, Bruker Optics, Ettlingen, Germany) operating in line-scan mode was used to examine the residual dentin after caries excavation (n = 8 per group). To standardize the areas of measurements, a straight line was drawn from the mesial to the distal outline of the excavated cavity AB as shown in (Fig. 2). Another line (X) descends from the center toward the cavity floor representing the 1st reference point (X1). Two further lines were drawn from X bisecting the 90° angles as 2nd and 3rd reference points (X2 and X3). Six points were scanned by Raman spectroscopy in each sample, 50 µm and 100 µm away from X1-X3, (Fig. 2). The peak value was the average of three readings at the superficial and deeper layers. For comparison with the remaining dentin substrates, a Raman control measurement was performed on sound reference dentin located 800 μm coronal to the dentin-enamel junction. Spectral acquisition utilized a 785 nm near-infrared diode laser coupled to a 400 lines/mm diffraction grating in a confocal Raman microscope (Senterra, Bruker Optics, Germany). An 20X Olympus objective (0.40 NA) produced a ≈ 5 μm diameter focal spot. Across a spectral range of 200–3600 cm^−1^, each site received 30 s exposures with an incident laser power of 100 mW. Post-processing entailed baseline correction of the obtained spectra (OPUS software, Bruker). Several key Raman peaks were examined to assess mineral and organic matrix components of dentin^15,16^, including: the v1 PO_4_^3−^ phosphate vibration at 960 cm^−1^, amide I protein band at 1650 cm^−1^, amide III at 1235 cm^−1^, and the pyrrolidine ring C-H bend in collagen at 1450 cm^−1^. Average peak intensities were calculated from 6 measurements equally spaced 50 and 100 μm from reference points (X1-X3) along the excavation interface (n = 6) in each sample. Collagen structural integrity was further evaluated using the amide III: pyrrolidine (1235 cm^−1^:1450 cm^−1^) absorbance intensity ratio, with values approaching ~ 1.0 suggesting an intact triple helical conformation^17,19^.

### Vickers microhardness

The microhardness of the remaining dentin was measured by Vickers microhardness tester (TH714, Obsnap Instruments Sdn Bhd, Selangor, Malaysia) using a square-based pyramid diamond-shaped indenter and 300 gf load for 15 sec^27^. Six indentations were made in each excavated cavity using the same points that were previously assessed by Raman spectroscopy (Fig. 2). The Vickers hardness number (VHN) was the average of three indentation per cavity at each area 50 µm and 100 µm from the excavated margins. The values were recorded automatically using the manufacturer’s software.

### Scanning electron microscopy- energy dispersive X-ray spectroscopy (SEM–EDX)

To assess the ultrastructural morphology, five representative specimens per group were sectioned horizontally through the excavated surfaces with a low-speed water-cooled diamond saw microtome (Isomet 1000, Buehler, IL, USA), and cleaned ultrasonically. The excavated surfaces were coated with carbon for conductivity and scanning electron microscopy (SEM) was performed using a field emission instrument (Inspect F50, FEI, Eindhoven, Netherlands) with 30 kV accelerating voltage, and three sequential magnification powers (1500X, 3000X, and 6000X). The qualitative assessments focused on the texture of the remaining dentin, the presence or absence of smear layer, the patency of the dentinal tubules. For further quantitative evaluation, energy dispersive X-ray spectroscopy (EDX) was used to determine the calcium and phosphorus atomic percentages and calcium:phosphorus ratios of the residual dentin, in which the mean values across three sites were compared to sound reference dentin.

### Statistical analyses

The statistical analyses were performed using SPSS software version 25 (IBM, Chicago, IL, USA). Shapiro–Wilk test was used to evaluate the normality of data distribution. The excavation time (min), DIAGNOdent readings and the Ca/P ratios were analyzed by One-way ANOVA test, while the Raman peak intensities (A.U.) and VHN were assessed by Multivariant ANOVA (MANOVA). Both tests were followed by Tukey post-hoc multiple comparisons (p > 0.05).

### Ethical approval

The Research Ethical Committee of the College of Dentistry University of Baghdad granted the ethical approval for the present study (Ref. No. 754, 28/12/2022). All procedures complied with the relevant guidelines and regulations of the local institutional committee in accordance with the 1964 Declaration of Helsinki and its subsequent amendments or comparable ethical standards. The authors confirm that informed consent was obtained from all patients and/or their legal representatives for all extracted teeth. The ethical standards were strictly followed in this study. The authors confirm that at each stage of the research: (1) no individual was exposed to any component of the materials or methods used in the research without being potentially harmed (2) the authors did not use any live plants or cytotoxic materials in this research (3) components or materials were not used in the research in a manner or concentration that would cause direct or indirect harm to the individuals conducting the research or to those involved in the various measurement procedures. (4) All materials used in the research have been used in a scientific, sound and accurate manner which includes the safety of persons and places in accordance with applicable local regulations and laws.

### Clinical relevance

The application of minimally invasive methods for caries removal can be considered as conservative alternatives to rotary excavation, to preserve the remineralizable tooth tissue providing a promising strategy for clinical practice.

## Results

### The excavation times

The rotary excavation method recorded the lowest caries removal time (6.9 ± 1 min, p < 0.001) among all methods, while the longer time was observed in Brix 3000 group (13.1 ± 1 min), with statistically no significant difference between the ultrasonic abrasion and laser ablation (11.2 ± 1, 10.5 ± 2 min, respectively, p = 0.80), Table 1.

**Table 1:** The mean caries excavation time (minutes), Diagnodent pen readings, and the calcium to phosphate ratios of the remaining dentin after each caries removal method and their corresponded sound dentin reference.

Groups (n = 10 per group)	Excavation time (minutes) (mean ± SD)	Diagnodent readings (mean ± SD)	Ca/P ratio (mean ± SD)	
			Residual dentin	Sound dentin
Rotary excavation	6.9 ± 1.2*	17.5 ± 1.7^b^	1.91 ± 0.05^c^^	2.04 ± 0.04
Brix 3000	13.1 ± 1.6	16.8 ± 1.2^b^	2.00 ± 0.06^c^	2.00 ± 0.05
Ultrasonic	11.2 ± 1.1^a^	15.9 ± 1.4^b^	2.23 ± 0.04*^^^	2.27 ± 0.05
Laser	10.5 ± 2.1^a^	13.8 ± 1.6*	1.99 ± 0.05^c^	2.21 ± 0.06

*The presence of statistically significant differences among caries excavation methods within each column.

Similar letters indicate that there were statistically no significant differences between methods.

^^^The presence of statistically significant differences in Ca:P ratios in each method from their correspondent sound dentin within each row (alpha level of 0.05).

### DIAGNOdent pen readings

The mean readings were ranged between 14 and 18 in the remaining dentin after all excavation methods, and the laser ablation recorded the lowest reading (p > 0.001), with statistically no significant differences between other techniques (p > 0.05), Table 1.

### The calcium to phosphorus (Ca:P) ratio

The Ca:P ratio of the remaining dentin was statistically significantly higher in the ultrasonic excavation group (2.27 ± 0.05) in comparison to all methods and the related sound reference (p < 0.05). The rotary excavation showed the lowest ratio (1.91 ± 0.05) among groups and from the corresponded sound reference (p = 0.012), Table 1.

### Biochemical analysis of the remaining dentin after caries removal

The relative Raman band intensities (Mean ± SD) with statistical correlations of the remaining superficial and deep dentin layers (50 µm and 100 µm) after caries removal are presented in Table 2. Multivariant ANOVA test showed the presence of statistically significant differences between groups at each area and between areas within the same group in all Raman peaks (p < 0.05). Further analysis using Tukey post-hoc test revealed that the phosphate band intensities were statistically significantly lower at the superficial remaining dentin layer (50 µm) after all excavation methods in comparison to their relative values at the deeper layer (100 µm) and their sound references (p < 0.001). The highest phosphate peak intensity was noticed in the rotary excavation in comparison to other methods that showed statistically no significant differences between them at both layers (p > 0.05), Table 2.

**Table 2:** Raman inorganic and organic peak intensities (mean ± SD) of the remaining superficial and deep dentin layers after different caries removal techniques.

	Raman peak intensities at 50 µm	Raman peak intensities at 100 µm	Raman peak intensities of reference points
Phosphate peak v1-PO (960 cm^−1^)			
Rotary excavation	2508.8 ± 296.2*	2849.6 ± 272.4*	3188.4 ± 288.8
Brix 3000	1967.0 ± 234.1^a^*	2338.2 ± 281.1^b^*	2970.5 ± 258.7
Ultrasonic	2055.3 ± 280.5^a^*	2521.5 ± 258.3^b^*	3016.9 ± 223.4
Laser	1929.2 ± 226.8^a^*	2451.5 ± 186.9^b^*	2826.9 ± 214.4
Amide I (1650 cm^−1^)			
Rotary excavation	25.4 ± 3.5^c^	23.8 ± 3.1^c^	22.1 ± 3.0^c^
Brix 3000	37.0 ± 4.5*	29.3 ± 5.7*	19.7 ± 3.8
Ultrasonic	50.1 ± 4.9^d^*	39.0 ± 4.9^e^*	21.2 ± 2.7
Laser	49.9 ± 3.6^d^*	41.1 ± 3.7^e^*	22.1 ± 3.1
Amide III (1235 cm^−1^)			
Rotary excavation	34.4 ± 4.0^f^	33.4 ± 7.0^f^	30.7 ± 3.5^f^
Brix 3000	45.0 ± 4.6^g^*	40.5 ± 4.6^g^*	30.0 ± 8.6
Ultrasonic	69.1 ± 4.5*	45.3 ± 3.3^g^*	29.1 ± 2.7
Laser	54.9 ± 5.4*	41.4 ± 4.7^g^*	29.8 ± 3.2
C-H bond of pyrrolidine ring (1450 cm^−1^)			
Rotary excavation	35.9 ± 2.5^h^*	32.4 ± 6.3^h^	30.4 ± 3.7^h^
Brix 3000	38.7 ± 4.0^j^*	34.8 ± 5.4^j^*	28.7 ± 4.3
Ultrasonic	48.8 ± 5.4*	40.1 ± 6.0^j^*	29.3 ± 5.8
Laser	41.3 ± 5.0^j^*	38.3 ± 5.3^j^*	31.7 ± 7.3

Similar letters indicate that there were statistically no significant differences between groups and areas.

*The presence of statistically significant differences (p < 0.05) in Raman peak intensities at the superficial and deep dentin layers from their correspondent sound dentin within each row. Multivariant ANOVA test and Tukey’s post-hoc tests (alpha level of 0.05).

For the organic components (Amide I, Amide III, and C-H bond of pyrrolidine ring), the Raman peak intensities at 1650 cm^−1^, 1235 cm^−1^ and 1450 cm^−1^, respectively, were higher in the superficial layers of the remaining dentin after all MI caries removal methods in comparison to their alternative values at the deeper dentin layers and sound reference (p < 0.001). Whilst, the rotary excavation showed the lowest organic content (p < 0.001) which were statistically not significant differences between areas and from sound references (p > 0.05). The highest organic peak intensities were observed in the ultrasonic and laser groups at both layers (p < 0.001). However, the differences in values of Amide III and C-H bond of pyrrolidine ring were statistically not significant from Brix 3000 (p > 0.05) at the superficial and deep dentin layers.

**Figure 3:**
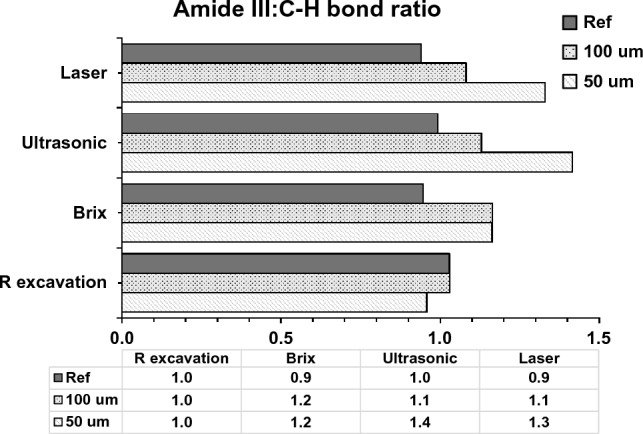
Means of dentin collagen integrity ratios (Amide III: C-H pyrrolidine ring, 1235 cm^−1^:1450 cm^−1^) which ranged from 0.9 -1.4 after all excavation methods and close to sound dentin references (1.0).

Regarding the collagen integrity, the absorbance ratio of amide III bands at 1235 cm^−1^ to the pyrrolidine ring at 1450 cm^−1^, ranged between 0.9 and 1.4 in all groups which are close to sound dentin reference (1.0). This might indicate the lack of collagen denaturation in the remaining dentin after all excavation techniques. The higher ratios were seen in the ultrasonic and laser groups due to the high absorbance band of the amide III bands at 1235 cm^−1^ (amide III), (Fig. 3).

### Vickers microhardness

**Figure 4:**
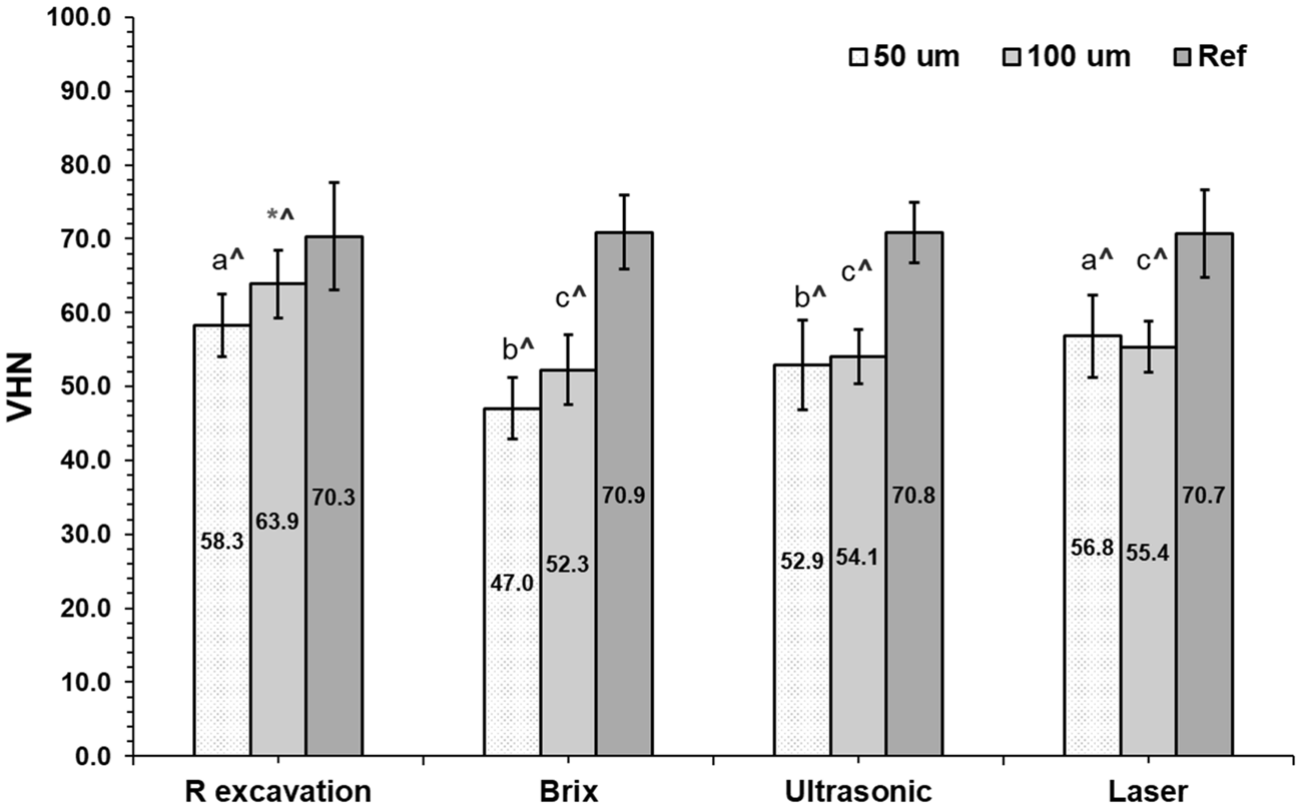
Mean Vickers hardness number of the remaining dentin after different caries removal techniques. Similar letters indicate that there were statistically no significant differences between the excavation methods at each area. (*) The rotary excavation method exhibited the highest value from all methods (p < 0.05) at the deep dentin layer. (^) the presence of statistically significant differences in each method from their corresponded reference sound dentin. Multivariant ANOVA test and Tukey’s post hoc tests (alpha level of 0.05).

At the superficial remaining dentin, the rotary excavation and laser ablation recorded the highest VHN among groups (p < 0.001) with statistically no significant difference between Brix 3000 and ultrasonic application (p = 0.061). Whilst, at the deeper layer, the surface hardness was the highest in the rotary method (p < 0.001) with statistically no significant differences between the MI methods (p = 0.999). Generally, the VHN values of the remaining dentin after all caries removal methods were statistically significantly lower than their corresponded sound dentin references (Fig. 4).

## Discussion

The ideal caries-excavation method must have the ability to selectively remove the irreversibly damaged tissue, while preserving the potentially remineralizable dentin that can be healed and repaired by the dentin-pulp complex or the use of bioreactive materials^5,24^. However, this is difficult to be achieved clinically, even when the currently available caries excavation techniques are claimed to be specific in removing the infected tissues without sacrificing sound or potentially remineralizable tissues. This study demonstrated the effectiveness and efficiency of three MI caries removal techniques (Brix 3000, ultrasonic, and laser ablation) in preserving chemomechanically and morphologically favorable residual dentin compared to the conventional rotary excavation.

The excavation endpoint was determined visually and by tactile examination using a sharp dental explorer. However, the operators’ experience and variations in the characteristics features of lesions may influence the clinical judgment of the remaining tissues. Even though, these methods can sufficiently evaluate the residual dentin clinically when combined with caries detection aids like dyes or laser fluorescence^28,29^. Further examination was accomplished by applying DIAGNOdent pen that can quantify the fluorescence induced by the bacterial metabolites in infected tissues^30^. The mean DIAGNOdent readings were comparable between methods (16–17.5), but the laser ablation recorded the lowest reading among methods (p < 0.05, Table 1), which inconsistent with Eberhard et al. (2005)^31^ who observed a two to three-fold increase in the fluorescence after laser excavation due to the dehydration of dentin that induces higher fluorescence. Although DIAGNOdent readings cannot discern between active and arrested lesions, as in Maillard reaction that mediates dentin discoloration shows high fluorescence independent of the bacterial levels^32^. Still, combining DIAGNOdent with visual and tactile inspection methods can sufficiently determine the excavation endpoint when utilized by experienced clinician^28,33^.

In agreement with the literature^13,34,35^ the bur excavation was the quickest among all techniques (7 ± 1 min). This is attributed to lack of sensitivity of tactile feedback during tooth cutting which leads to gross and rapid removal of carious and non-carious tissues with less control over the whole process. In contrast, the chemomechanical agent was the slowest among techniques (13 ± 1.6 min, p < 0.05). This attributed to the multiple applications of the gel until complete caries removal, added to the variations in the size and activity of the carious lesions which are difficult to be controlled or expected. Even though, the CMCR agent is widely accepted since it reduces pain in fearful and young patients^36^ with a reported selectivity^6^ in removing the damaged carious tissues while preserving sound dentin. The time taken by Er,Cr:YSGG Laser was longer than the rotary excavation, which was comparable to the ultrasonic caries removal (≃11 min). This consistent with Johar et al. (2019)^37^ who reported a longer excavation time for the Er,Cr:YSGG Laser (6 W power, 55.5 mJ/cm^2^ pulse energy, and 4 Hz frequency) than rotary excavation in primary teeth in an in vivo study. Celiberti et al. (2006)^38^ also reported a double-time needed to remove caries in deciduous molars when the Er:YAG laser (2.94 µm wavelength, 200 mJ pulse energy, and 4 Hz frequency) was applied in comparison to bur excavation. However, Shigetani et al. (2002)^39^ found no significant difference in the excavation time between both methods. Even though, the use of laser can reduce pain and stress during cavity preparation and dentin loss in comparison to rotary method^31^.

Biochemically, the mineral component in the residual dentin represented by the characteristic phosphate (*v*1 PO_4_^3−^) Raman peak at 960 cm^−1^, which the most intense peak in dental hard tissue. It was significantly reduced (p < 0.05) after all excavation methods in comparison to sound dentin which also varied in different depths based on the degree of tissue demineralization indicating the presence of CAD as reported in previous studies^17,40^. The highest phosphate peak intensity was noticed in the bur-excavated surfaces which is located within more highly mineralized dentin. This supports the non-selectiveness of this technique that has been proven in previous studies^41,42^. Whilst, in the minimally-treated dentin (Brix 3000, ultrasonic, and laser), the overall phosphate content was initially lower than sound dentin, but still, they contain more residual mineral in comparison to the reported values for the CAD in a previous study^17^. This relatively high mineral level after all excavation methods might affect the subsequent mineral deposition rate and distribution that will help in situ generation of prenucleation clusters, succeeding further dentin remineralization^43^.

The intensity of the organic functional groups can quantitatively indicate the biochemical alteration in dentin during carious process which directly affect tissue repair and adhesion. The dentin collagen matrix features were found at 1650 cm^−1^(Amide I), 1235 cm^−1^ (Amide III), and at 1450 cm^−1^ (C-H bond of pyrrolidine ring). These bands intensities were higher in the superficial layer of the minimally-excavated dentin in comparison to bur-excavated and sound dentin. This might indicate that the residual dentin in the minimally excavated cavities contains demineralized tissues with high organic contents resulted from the degradation of extracellular organic matrix after dissolution of the hydroxyapatite. This fact is well supported in previous studies^17,44,45^ that observed higher organic contents in CAD in comparison to sound dentin. The intensity of Amide I was the highest in the ultrasonically and laser excavated-surfaces (p < 0.001), indicating the presence of structural alterations in the remaining collagen^46^. This might support the selective ability of both technique as this altered organic matrix can regulate the growth and maturation of apatite crystals and thus the mineralization process^40^. The ultrasonic does not physically cut the dentin but abrades by a diamond-coated tip oscillating at 28 kHz vibration frequency. This oscillation was transformed into vibration at the dentin surface that may have a compacting effect on the carious dentin giving false indication for the clinical excavation endpoint. The chemical dissolution of carious tissues by Brix 3000 also maintained high organic content in the residual dentin especially the Amide III and the C-H bond of pyrrolidine ring in comparison to rotary excavation and sound dentin (p < 0.001). This referred to the action of papain enzyme that breakdown the partially degraded collagen and help in the disintegration of fibrin mantle generated by the carious process while preserving the unimpaired collagen fibrils. However, it is difficult to clearly distinguish between the dissolution effect of the gel from the assisted mechanical removal. The preservation of the organic components in the minimally treated surfaces can provide a suitable scaffold for the biomimetic dentin remineralization and induce tissue healing^47^. This contradicts with Pai et al. (2009)^48^ who found no significant difference in the organic contents of the remaining tissues between rotary and Carisolv, which might be due to the chlorination effect of the chloramine T that may dissolve sound and demineralized tissues. The ratio of the intensity of Amide III to the C-H bond of pyrrolidine ring elucidates the integrity of the collagen fibril with a triple helical structure that may undergo micro-structural changes during the caries process if the ratio below 0.8^17,19^. The ratios in the remaining dentin after all excavation methods ranged between 0.9 and 1.3 which means that even with the presence of changes in the organic structure of dentin, the ratio was close to that reported for CAD and sound dentin (1.0), as shown in Fig. 3.

The bur-excavated tissues exhibited higher hardness values, however, at the superficial layer, the value was comparable to the laser ablation (p > 0.05), which was significantly increased (p > 0.001) in the deeper dentin layer. This might be attributed to the higher mineral contents in the residual dentin represented by the higher Raman phosphate intensity and the higher Ca and P atomic % (12.2, 6.6% respectively) even if the Ca:P ratio was the lowest among groups, table (1). This supports the non-selectiveness of this technique in comparison to the other used method, especially the chemomechanical caries removal technique (Brix 3000 group) that showed the lowest hardness values. Hamama et al. (2013)^42^ reported reduced hardness values of the residual dentin after using Carisolv and Papacarie in comparison to rotary excavation. This attributed to the chlorination effect of chloramine-T in both gels which decomposes the degenerated collagen facilitating its removal and can induce softening of the remaining dentin. Whilst, Lima Santos et al. (2020)^34^ did not find any significant difference in microhardness between the CMCR agents (Brix 3000 and Papacarie Duo) and rotary excavation. The hardness of the remaining tissues in Brix 3000 group was similar to the ultrasonic and laser methods (p = 0.999) that also showed reduced values as compared to bur excavation and sound dentin at the deeper layer (p < 0.001). This indicates the presence of partially demineralized tissues left after caries removal, as the hardness and elastic modulus of CAD is lower than sound dentin, because of the reduced number and size of the apatite crystals in the intertubular dentin after tissue demineralization^16,17,43^. However, the Ca/P ratios in all groups are comparable to sound dentin (2.0–2.2) which falls within the reported ratios^42,49^. Sakoolnamarka et al. (2005)^50^ found the presence of a correlation between the Ca:P ratio and microhardness in dentin using an Ultra-Micro-Indentation System. However, this cannot be applicable in the present study as the ultrasonic-excavated dentin recorded the highest Ca:P ratio (2.27 ± 0.05) than rotary excavation (p = 0.003), but the atomic percentages of Ca and P ions were 50% lower (6.9, 3.1%, respectively) associated with lower hardness value (p < 0.001).

**Figure 5:**
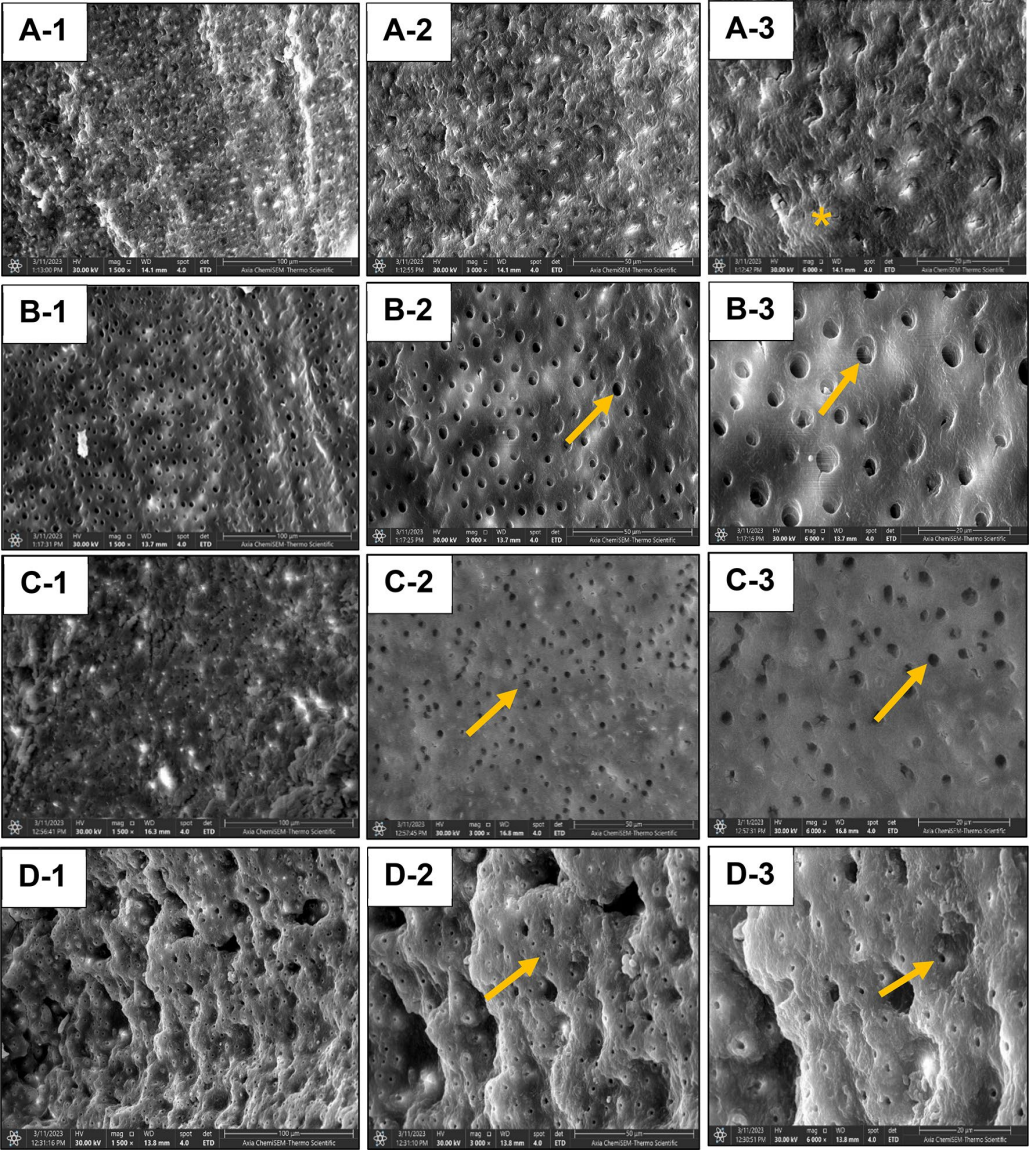
Scanning electron micrographs of the remaining dentin after caries excavation using rotary excavation (**A**), Brix 3000 (**B**), Ultrasonic abrasion (**C**), and Laser ablation (**D**). Three magnifications for each were used; 1500x, 3000x, and 6000x (1, 2, and 3, respectively. At higher magnification (2 and 3), both Brix and Ultrasonic groups produce smooth dentin surfaces with less amount of tissue debris that was clear rotary excavation with noticed rough surfaces. More patent dentin tubules (yellow arrow) were observed in Brix 3000 and ultrasonic excavation with exposed collagen networks in B-3. Whilst, in the bur-prepared cavity there is an evidence of marked smearing (yellow asterisk) and tubule orifice occlusion. SE micrograph of the cavity prepared by laser showing wavy and irregular surface with patent tubular orifices.

The minimally caries excavation techniques produced smooth dentin free from smear layer, leaving patent dentinal tubules which might facilitate the infiltration of adhesive resin into intertubular dentin. This agreed with previous studies^51,52^ regarding the sonic abrasion (Fig. 5C-2 and C-3). For the Brix 3000, the results are consistent with Hossain et al. (2003)^49^, but disagreed with Hamama (2013)^42^ and Al-Badri et al. (2023)^53^, who observed the presence of amorphous layer of cutting debris obliterated the dentinal tubules resulted from crushing of the excavated tissue by spoon excavator suggesting that the mechanical action is more relevant than the chemical effect. In agreement to a previous study^6^, the laser-ablated surfaces showed a wavy irregular surface with rugged appearance free from smear layer associated with patent dentinal tubules which might be attributed to the water flow that allows proper cleansing of the laser-ablated surfaces. This might enhance the bonding to adhesive restorations combined with the etching effect of the dental laser^15^.

In contrast, the rotary excavation technique produced rough dentin surface with a noticeable smearing accompanied which might interfere with adhesion, wetting, and the penetration of the adhesive-based restorations when used without preconditioning the substrate^54^. However, the presence of such micro-irregularities might increase the surface area for micromechanical retention. This coincides with Hamama (2013)^42^ and Prabhakar et al. (2018)^6^ who observed the presence of well-formed smear layer with occluded dentinal tubules. Although, leaving the CAD after caries excavation is highly recommended following the minimally -invasive concept, but the presence of a higher degree of collagen exposure within a thick organic-enriched smear layer might affect the hybridization with adhesive resins^55^ which necessitates further investigations.

However, the rate of caries progression may affect the status of dentinal tubules, as active lesions tend to have more patent tubules than arrested lesions^40^. This is difficult to predict with a possibility for chronic development that can alter the morphology of the dentinal tubules. In addition, the dentin is a hydrodynamic biological substrate that exhibits structural changes due to the physiologic processes over time, added to other factors such as, patients’ age, lesion depth and pulp response^40^.

Accordingly, the proposed hypotheses in this study were rejected as the use of Brix 3000, ultrasonic and laser ablation exhibited statistically significant differences in the chemomechanical properties of the residual dentin from bur excavation method and sound dentin. Despite the longer excavation time, they are capable of maintaining potentially mineralizable tissues with enhanced morphological features for subsequent bonding to restorative materials. However, the impacts of these caries removal techniques on the pulp health in deeper lesions, and their potential benefits for shallower lesions where dentin bonding is crucial, which necessitate further extensive in vitro and clinical studies to extrapolate these findings.

## Conclusion

The rotary technique achieved the shortest excavation time, while Brix 3000 gel showed the longest time. At a biochemical level, the bur-excavated dentin contains the highest mineral phosphate and lowest organic contents, conferring higher hardness approaching that of sound dentin. This aligns with non-selective removal of both infected and affected tissues. Alternatively, the MI techniques exhibited lower phosphate but higher organic matrix, with reduced tissue hardness especially in deeper dentin layers, indicating the conservation of partially demineralized tissues. This was associated with smooth dentin surfaces free from smear layer with patent dentinal tubules. Thus, the study supports the implantation of the MI caries removal methods as conservative alternatives to rotary excavation, with promising translational potential for preserving the repair capacity of the dentin-pulp complex after clinical caries removal.

## Data Availability

The data that support the findings of the current study are available from corresponding author upon reasonable request.
